# Human Basal and Suprabasal Keratinocytes Are Both Able to Generate and Maintain Dermo–Epidermal Skin Substitutes in Long-Term In Vivo Experiments

**DOI:** 10.3390/cells11142156

**Published:** 2022-07-09

**Authors:** Luca Pontiggia, Akshay Kumar Ahuja, Hesham Kamaleldin Yosef, Dominic Rütsche, Ernst Reichmann, Ueli Moehrlen, Thomas Biedermann

**Affiliations:** 1 Tissue Biology Research Unit, Department of Pediatric Surgery, University Children’s Hospital Zurich, Wagistrasse 12, 8952 Schlieren, Switzerland; ahujaakshay@gmail.com (A.K.A.); dominic.ruetsche@kispi.uzh.ch (D.R.); ereichmann55@gmail.com (E.R.); ueli.moehrlen@kispi.uzh.ch (U.M.); thomas.biedermann@kispi.uzh.ch (T.B.); 2 Children’s Research Center, University Children’s Hospital Zurich, 8032 Zurich, Switzerland; 3 Faculty of Medicine, University of Zurich, 8091 Zurich, Switzerland; 4 microPhotonX GmbH, 82327 Tutzing, Germany; hesham.yosef@microphotonx.com; 5 Department of Pediatric Surgery, University Children’s Hospital Zurich, University of Zurich, 8032 Zurich, Switzerland; 6 Zurich Center for Fetal Diagnosis and Treatment, 8032 Zurich, Switzerland

**Keywords:** keratinocyte, stem cells, regeneration, homeostasis, interfollicular epidermis, integrin

## Abstract

The basal layer of human interfollicular epidermis has been described to harbour both quiescent keratinocyte stem cells and a transit amplifying cell population that maintains the suprabasal epidermal layers. We performed immunofluorescence analyses and revealed that the main proliferative keratinocyte pool in vivo resides suprabasally. We isolated from the human epidermis two distinct cell populations, the basal and the suprabasal keratinocytes, according to the expression of integrin β4 (iβ4). We compared basal iβ4^+^ or suprabasal iβ4^−^ keratinocytes with respect to their proliferation and colony-forming ability and their Raman spectral properties. In addition, we generated dermo–epidermal substitutes using freshly isolated and sorted basal iβ4^+^ or suprabasal iβ4^−^ keratinocytes and transplanted them on immuno-compromised rats. We show that suprabasal iβ4^−^ keratinocytes acquire a similar proliferative capacity as basal iβ4^+^ keratinocytes after two weeks of culture in vitro, with expression of high levels of iβ4 and downregulation of K10 expression. In addition, both basal iβ4^+^ and suprabasal iβ4^−^ keratinocytes acquire authentic self-renewing properties during the in vitro 3D-culture phase and are able to generate and maintain a fully stratified epidermis for 16 weeks in vivo. Therefore, against the leading dogma, we propose that human suprabasal keratinocytes can retro-differentiate into true basal stem cells in a wound situation and/or when in contact with the basement membrane.

## 1. Introduction

Keratinocytes represent 90–95% of the total cell population in the human interfollicular epidermis (IFE) [1]. They are arranged in interactive layers, each of which contains cells at consecutive stages of differentiation [2]. The IFE is constantly regenerated by the proliferation of self-renewing keratinocytes, a process which have been studied over several decades and gives insight into the biological mechanisms that maintain the epidermis throughout life [3,4,5,6,7].

Nevertheless, identity and localization of self-renewing keratinocytes in the IFE are still largely debated [8]. Early research carried out on human and cynomolgus monkey skin sections revealed the presence of both basal- and suprabasal-dividing keratinocytes under homeostatic conditions [9,10,11]. However, later mouse studies contrasted these findings by showing that only the keratinocytes of the basal layer during homeostasis and the epithelial cells of the hair bulge have the ability to regenerate the IFE during wound repair [12,13]. Accordingly, label-retaining keratinocytes were shown in mice to reside only in the basal layer of the epidermis, from where they regenerate the entire epidermis [14].

In summary, these reports accredited two main models [15]: basal unipotent stem cells (SC) either (1) undergo asymmetric cell divisions producing a basal SC and committed spinous daughter cell which replenish the overlying epidermal layers [16,17,18] or (2) give rise alternately to basal SCs or to basal transit amplifying cells, which are committed progenitors that in turn generate differentiated cells which migrate into the upper epidermal layers [19,20,21]. Subsequent studies showed that both mechanisms can partially coexist at the same time [22,23,24]. However, these hypotheses are based on studies in mice and may not be applicable to the human skin [8].

Our investigation systematically characterizes the biological and biochemical properties of two distinct, however, adjacent, populations of keratinocytes in human skin. Additionally to common immuno-histochemical methods, we implemented Raman spectroscopy [25] as a non-invasive, label-free, and highly selective technique which has been used in many biomedical applications [26,27,28] to analyse the chemical structure of the isolated keratinocyte populations. We revealed that both basal and suprabasal human keratinocytes possess self-renewing properties and are able to generate and maintain a fully stratified epidermis under experimental conditions for 16 weeks. Since the proliferative population mainly resides in the first suprabasal epidermal layers, we propose a model in which the maintenance of the adult human IFE during homeostasis is predominantly achieved by the proliferation of actively cycling suprabasal keratinocytes, which, therefore, retain and exercise the self-renewing capacity at least during wound healing.

## 2. Materials and Methods

### 2.1. Histological and Immunofluorescence Staining

Histological haematoxylin/eosin and immunofluorescence staining on tissue sections are described in detail by Biedermann et al. [29]. All the used antibodies were specific for human antigens (see also the supplementary table of marker abbreviations and acronyms). From Dako (Baar, Switzerland): K10 (clone: DE-K10, 1:100), K19 (clone RCK108, 1:100); from SantaCruz (Labforce AG, Nunningen, Switzerland): Laminin 332 (clone: P3H9-2, 1:100); from Spring Bioscience (Pleasanton, CA, USA): K15 (clone: SPM190, 1:50); from Progen (Heidelberg, Germany): K2e (clone Ks 2.342.7.4, 1:100); from Chemicon (Millipore AG, Zug, Switzerland): K16 (clone LL025, 1:100); from Zymed (Invitrogen, Basel, Switzerland): Desmoglein 1 (clone 27B2, 1:50), Desmoglein 3 (clone 5G11, 1:200); from BD Pharmingen (Basel, Switzerland): Ki67 (clone B56, 1:200); CD104 (integrin β4, clone 7/CD104, 1:300); CD49f (integrin α6)-Phycoerythrin (clone GoH3, 1:10); from Biotechne (Zug, Switzerland): K15-Alexa Fluor 405 (clone LHK15, 1:50); Ki67-Alexa Fluor 594 (clone 8D5, 1:50); CD104 (integrin β4)-Alexa Fluor 488 (clone 422325, 1:100). As secondary antibodies, we used Alexa Fluor 488, Alexa Fluor 568 or Alexa Fluor 647-conjugated polyclonal donkey IgG directed to mouse immunoglobulins (Abcam, Cambridge, UK). For double immunofluorescence staining, some of the primary antibodies were pre-labeled with Alexa 555-conjugated polyclonal goat F(ab’)2 fragments, according to the instructions of the manufacturer (Zenon Mouse IgG Labeling Kit, Molecular Probes, Invitrogen, Basel, Switzerland). The counterstaining of cell nuclei was performed with Hoechst 33342 (Sigma-Aldrich, Buchs, Switzerland). Pictures were taken with a DS-Ri1 digital camera connected to a Nikon Eclipse TE2000-U inverted microscope. The device is equipped with Hoechst-, FITC-, TRITC- and APC-filter sets (Nikon AG, Egg, Switzerland; Software: NIS-Elements BR version 3.22.11).

### 2.2. Cell Isolation and Culture

Human skin samples were obtained from the Department of Surgery of the University Children’s Hospital in Zurich. Parents or patients gave informed consent. This study was conducted according to the Declaration of Helsinki Principles and after permission by the Ethics Commission of the Canton Zurich (BASEC Request No. 2018-00269).

Human epidermal keratinocytes and dermal fibroblasts were isolated and cultured according to Pontiggia et al. [30]. Briefly, the biopsies were digested overnight in 12 U mL^−1^ dispase (BD Biosciences, Allschwil, Switzerland) dissolved in Hank’s balanced salt solution containing 5 mg mL^−1^ gentamycin (both Thermo Fisher Scientific AG, Basel, Switzerland) at 4 °C. The epidermis and dermis were subsequently separated using forceps. The epidermis was digested in 1% trypsin and 5 mM EDTA for 3 min at 37 °C (Thermo Fisher Scientific). Epidermal cells were passed through a 100 µm cell strainer (ClearLine, Milian AG, Boswil, Switzerland), washed in phosphate buffered saline (PBS, Thermo Fisher Scientific) and resuspended in serum free keratinocyte medium CT57 (CellnTec, Bern, Switzerland) containing bovine pituitary extract, epidermal growth factor and the addition of 5 µg mL^−1^ gentamycin. For keratinocyte selection, the epidermal cells were cultured on bovine collagen I (Symathèse, Chaponost, France) coated culture flasks. For gaining fibroblasts, the dermal tissue was further digested in 2 mg mL^−1^ collagenase blend F (Sigma-Aldrich Chemie GmbH, Buchs, Switzerland) for 1 h at 37 °C. After mechanical disruption by pipetting, lysis of red blood cells in RBC lysis buffer according to the instruction of the manufacturer (Sigma) and washing in PBS, the dermal fibroblasts were plated on uncoated culture flasks and grown in DMEM containing 10% foetal calf serum (FCS), 4 mM l-alanyl-l-glutamine, 1 mM sodium pyruvate and 5 mg mL^−1^ gentamycin (all Thermo Fisher Scientific). The medium was changed every 2–3 days and cells were passaged when approximately 80–90% confluence was reached.

### 2.3. Ki67 Quantification

For quantification of the number of basal- and suprabasal-cycling (Ki67 positive) keratinocytes, 30 × 10^3^ freshly isolated keratinocytes were concentrated onto a microscope slide by cytospin centrifugation (Histocom AG, Zug, Switzerland). After fixation for 3 min in aceton:methanol 1:1, the cells were stained with the Ki67 and K10 antibodies as mentioned above. For each sample (*n* = 5), we analyzed three different microscopic frames which were taken randomly. We calculated which percentage of the total number of Ki67 positive cells was Ki67-single positive (cycling basal cells) or Ki67/K10-double positive (cycling suprabasal cells). Means, standard deviations, and significances (unpaired *t* test) were calculated using GraphPad Prism (Prism 8.0.0, GraphPad Software Inc., La Jolla, CA, USA). Significance was set at an alpha-level of 0.005. In few cases, we stained additionally for K15 and iβ4 to double-check the identity of basal cells and for illustrative purposes.

### 2.4. Fluorescence Activated Cell Sorting (FACS) and Analysis

After washing twice with PBS, freshly isolated epidermal cells were resuspended at 10 × 10^6^ cells per ml in cell culture medium CT57 containing the iβ4-Alexa Fluor 488 antibody (see above). In some experiments, iα6-PE or Zenon pre-labelled K10 antibodies were used (see above). In addition, the Zombie Aqua Viability Dye (Biolegend, Lucerna Chem AG, Lucerne, Switzerland) was added according to the instruction of the manufacturer. The cells were incubated on ice for 20 min. Before centrifugation, the cell suspension was adjusted to 4 mL with PBS and underlaid with 1 mL fetal calf serum (Invitrogen). Finally, the cells were resuspended in CT57 and transferred into a centrifugation tube through a 50 µm cell strainer cap (Corning, Wiesbaden, Germany). Flow cytometric analysis and sorting was performed at the Center for Microscopy and Image Analysis of the University of Zurich on a FACS Aria III (BD Biosciences, Allschwil, Switzerland) equipped with 405 nm (violet), 488 nm (blue), 561 nm (yellow/green) and 635 nm (red) lasers and using the 85 µm nozzle. Sorted cells were collected in CT57 medium with 50% FCS, washed once in PBS, counted and either plated on collagen I-coated dishes and further cultured in CT57 medium, or directly seeded on dermal substitutes (see Section 2.8).

### 2.5. Proteomics Analysis of Sorted Basal and Suprabasal Keratinocytes

100,000 freshly isolated and sorted keratinocytes (iβ4^+^ or iβ4^−^) were washed twice in PBS and stored at −80 °C until further processing. Cells from 4 different donors were collected. Peptide samples were prepared according to established protocols [31], with slight modifications. Briefly, proteins were extracted by incubating thawed cell pellets in 100 μL of 100 mM Tris/HCL (pH 8.2) for 5 min at 95 °C on a Thermoshaker at 1000 rpm, vortexing and spinning down. Protein concentration was determined using the Qubit™ Protein Assay Kit (Thermo Fisher Scientific) and 50 μg of protein was used for further processing using filter-aided sample preparation (FASP) with trypsin digestion overnight (1:50 ratio of trypsin to protein). Following FASP, tryptic peptides were centrifuged (14,000× *g*, 20 min, RT) and the filtrate was collected. The filtrate was then acidified with 5% trifluoroacetic acid (TFA) to a final concentration of 0.5%. After clean-up, samples were resuspended in 3% acetonitrile and 0.1% formic acid then cleaned up using C18 stage tips (Pierce), following manufacturer’s instructions. Samples were analyzed on a Q-Exactive HF Orbitrap LC-MS/MS mass spectrometer (Thermo Fisher Scientific). The device was run in data-independent acquisition (DIA) mode using XCalibur control software (Thermo Fisher Scientific). Automated quality control was conducted every 3 samples. Data and sample annotation was done using b-fabric (Available online: https://fgcz-bfabric.uzh.ch/bfabric/ (accessed on 15 June 2020). Identification was carried out using MASCOT (Matrix Science), using decoys. Protein quantification of the DIA data sets was carried out using Spectronaut (Biognosys AG, Schlieren, Switzerland). Filters for the generation of volcano plots are set to ≥1.5 absolute fold change. All *p*-values are corrected for multiple testing [32].

Raw mass spectrometry proteome data and search results have been deposited to the ProteomeXchange Consortium (http://proteomecentral.proteomexchange.org) (accessed on 10 June 2022) via the Proteomics Identifications Database (PRIDE) partner repository with the data set identifier PXD034543.

### 2.6. Colony Formation Assay

The colony-forming assay was performed according to Pontiggia et al. [33], with some modifications: 200 freshly isolated or 15 days-cultured cells (unsorted or sorted iβ4^+^ and iβ4^−^), were seeded on mitomycin (Sigma) treated 3T3-Swiss albino mouse feeder fibroblasts (ATCC Number: CCL-92) in six-well culture plates. The experiment was run in triplicate for each cell type for 8 different donors. After 10 days, single colonies were visible. The colonies were washed in PBS, fixed in aceton:methanol (1:1) for 5 min and stained in 0.2% trypan-blue solution (Sigma) for 5 min. After thorough washing, pictures of the plates were taken by standard light microscopy in a G:BOX Imaging System (Syngene, Cambridge, UK), the colonies were manually counted with the *Cell Counter* plugin of Fiji [34] and statistically analyzed: the number of colonies formed per 100 seeded cells is reported in Figure 4. Means of 8 experiments, standard deviations, and significances (unpaired *t* test) were calculated using GraphPad Prism. Significance was set at an alpha-level of 0.05.

### 2.7. Raman Microscopic Measurements

Raman measurements are implemented using Raman microscopic laser trapping system (BioRam^®^, microPhotonX GmbH, Tutzing, Germany) with an inverted microscopic setup. A diode laser of 785 nm wavelength and 80 mw power (TOPTICA Photonics AG, Graefelfing, Germany) is used for Raman excitation. All Raman scattered photons are collected by a 60-fold water immersion objective (1.1 NA, 0.2 WD) (Olympus, Hamburg, Germany) with correction collar set to 0.17 mm. The detection of the Raman scattered photons is achieved using a diffraction grating and a Charge-Coupled Device detector (CCD, Andor, Belfast, UK). 25 μL of PFA-fixed cells in PBS buffer were pipetted into a sterile μ-channel Slide with a 0.17 mm thick borosilicate glass bottom. 60 spectra were collected from the cytoplasmic domain of the cells. Cells were exposed to the laser for 15 s to collect a single Raman spectrum. Cells were trapped by the laser focal point during the measurements using optical trapping capability. Statistical analysis of Raman data: Processing of the spectral data and statistical analysis are performed using CT-RamSES software (microPhotonX GmbH, Tutzing, Germany). All Raman spectra were cropped to the spectral range of 350–1800 cm^−1^, which contains most of the biological-relevant spectral bands. This was followed by a baseline correction using an asymmetric least square fit, cosmic spikes were removed, and the spectra were smoothened with a median filter. Next, the spectra were interpolated to continuous wave numbers and normalized by implementing a unit vector normalization. Principal Component Analysis (PCA) was then applied on the datasets. PCA is a multivariate statistical analysis method used to reduce the dimensions of large data sets by transforming the large data variables into smaller numbers of components called Principal components (PCs). The first principal component (PC1) explains for the largest possible variance in the data set (most important variances), the PC2 explains the second most important, etc. PCA was performed under Python 3, using the scikit-learn package. PCA Score plots can illuminate the differences and similarities among samples, while the PCA Loadings display the Raman spectral differences that are used to compare the analysed samples. Hierarchical cluster analysis (HCA) was applied as an unsupervised statistical analysis and clustering method, to separate the Raman spectra based on the different spectral observations to different clusters. Similar to PCA, linear discriminant analysis (LDA) was applied to characterize differences between samples. However, LDA is a stronger tool for samples discrimination than PCA. In LDA, linear transformations of the variables to the new discriminant functions (LDs) are computed, in which the directions of the LDs are determined in the spectral space [35].

### 2.8. Organotypic Cultures

Organotypic cultures were prepared using a previously established transwell system consisting of 6-well cell culture inserts with membranes of 3.0 µm pore-size (BD Falcon, Basel, Switzerland) [30]. The collagen matrix was prepared according to the protocol of Costea et al. [36] with modifications. Briefly, 2.0 mL of bovine collagen type I (Symathèse), were added to a 0.6 mL neutralization buffer (0.32 M NaHCO_3,_ 0.15 M NaOH and 0.2 M Hepes, all from Sigma) and 0.4 mL DMEM containing 50 × 10^3^ fibroblasts. After polymerization (5 min at room temperature and two hours at 37 °C), the dermal substitutes were compressed to 1 mm thickness according to Braziulis et al. [37] and grown in DMEM/10% FCS for 7 days. Subsequently, 1.5 × 10^6^ freshly isolated and sorted keratinocytes in 1.5 mL CT57 were seeded onto each dermal equivalent. Triplicate wells were set up for each dermo–epidermal substitute. The constructs were cultured with DMEM in the lower chamber and CT57 in the upper chamber with daily medium change. After 7 days, the dermo–epidermal substitutes were ready for transplantation.

### 2.9. In Vivo Transplantation

Animal studies were approved by the local committee for experimental animal research (license number ZH90/15 and ZH45/19) and performed as previously published [29]. Ten weeks old immune-incompetent female crl:NIH-Foxn1rnu rats (Charles River, Freiburg, Germany) were prepared and anesthetized as described previously [38,39]. Skin substitutes were transplanted into full thickness skin defects created surgically on the back of the animals. To prevent wound closure from rat skin, custom made metal rings (2.5 cm in diameter) were sutured to the skin defects using non-absorbable polyester sutures (Ethicon, Johnson and Johnson, Zug, Switzerland). The wound was closed with multiple layers of protective wound dressing. Buprenorphin (s.c. 0.05 mg kg^−1^ body weight, TemgesicTM, Indivior AG, Baar, Switzerland) was applied for analgesia. Dressing changes and photographic pictures were made once a week. Rats were monitored for the entire length of the experiment. At week 16 post transplantation, animals were sacrificed, and transplants were excised and embedded either in paraffin or TissueTek (OCT compound, Sakura, Alphen aan den Rijn, Netherlands) for histological and immunofluorescence analysis. A total of 6 rats received a dermo–epidermal skin substitute formed with iβ4^+^ keratinocytes and 10 rats with iβ4^−^ keratinocytes.

## 3. Results

### 3.1. Suprabasal Keratinocytes Proliferate in the Human Interfollicular Epidermis under Physiological Conditions

**Figure 1:**
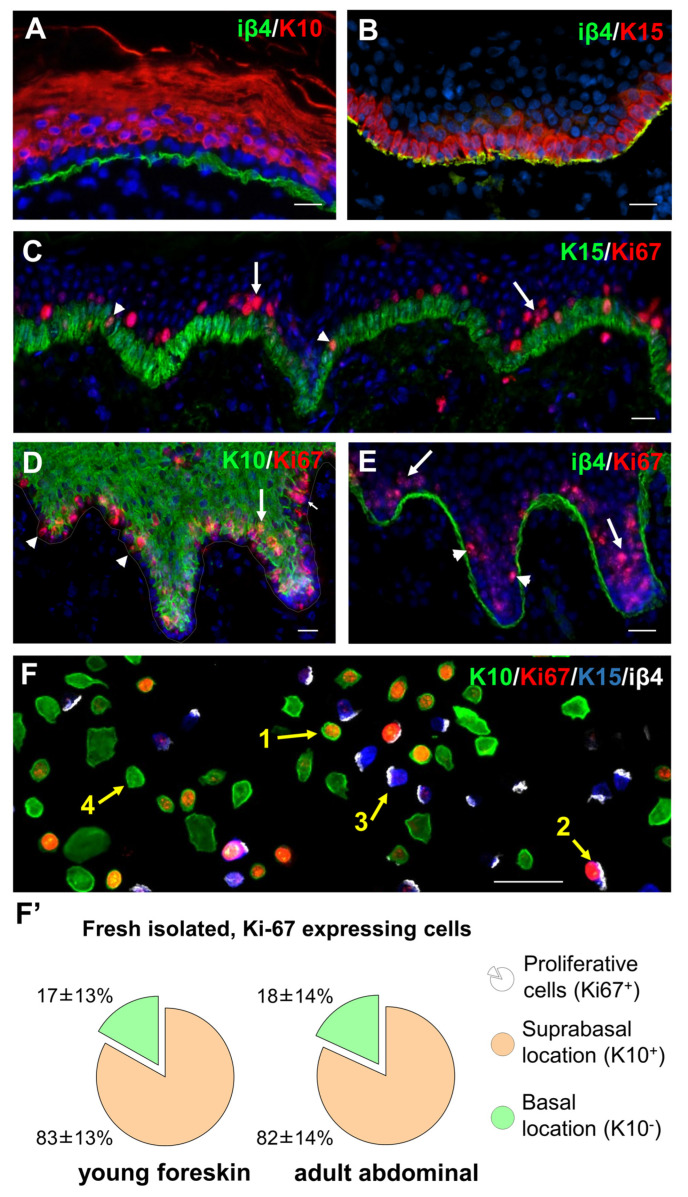
Suprabasal keratinocytes proliferate during homeostasis in human skin. Immunofluorescence double-staining of normal human abdominal skin cryosections with antibodies against (**A**) iβ4 (green) and K10 (red), (**B**) iβ4 (green) and K15 (red), (**C**) K15 (green) and Ki67 (red), (**D**) K10 (green) and Ki67 (red), (**E**) iβ4 (green) and Ki67 (red) show that cycling keratinocytes can be found almost exclusively in suprabasal layers. (**F**) Cytospin centrifugation of freshly isolated keratinocytes and staining for Ki67 (red) and K10 (green) to allow for quantification of proliferating cells and their assignment to the basal or suprabasal cell pool. In this picture, cells were stained additionally for K15 (blue) and iβ4 (white) to double-check the identity of basal cells. Yellow arrows point to four different cell types: proliferating suprabasal cells (1), proliferating basal cell (2), quiescent basal cell (3) and quiescent suprabasal cell (4). Single colour images and magnification can be seen in the Supplementary Figure S1. (**F’**) The statistical analysis of the original intra-epidermal localization of freshly isolated Ki67 positive keratinocytes from foreskin biopsies (*n* = 6) as well as from abdominal skin (*n* = 5). The pie charts show the mean values, the labels report mean values and standard deviations. The significance (unpaired *t* test) is given in both cases with *p* < 0.005. All scale bars: 50 μm.

**Figure 2:**
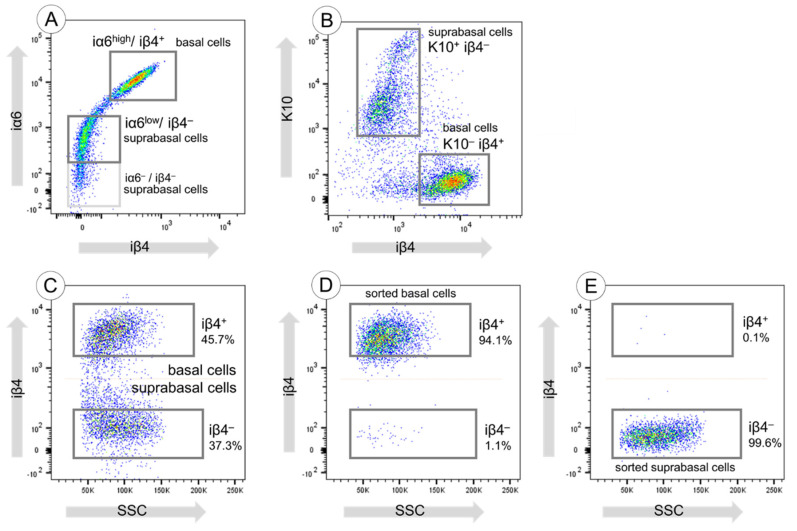
iβ4 expression discriminates basal and suprabasal keratinocytes. (**A**) Flow cytometry analyses of freshly isolated keratinocytes stained for iα6 and iβ4 revealed the correlation between the iα6^high^ and the iβ4^+^ population. (**B**) In the same way, co-staining of iβ4 and K10 demonstrated the inverse correlation between K10 and iβ4 expression which allowed the clear discrimination of suprabasal and basal cells. (**C**) FACS analysis and sorting of keratinocytes stained for iβ4 only. Two distinct populations were visible: basal iβ4^+^ and suprabasal iβ4^−^ keratinocytes. (**D**) Sorted iβ4^+^ population. (**E**) Sorted iβ4^−^ population.

To characterize the epithelial cells that are responsible for epidermal regeneration in homeostasis, we first sought to identify keratinocytes that are actively cycling in the normal human interfollicular epidermis. A clear discrimination between the basal and the suprabasal cell populations was achieved by immunofluorescence analyses of human skin sections. Figure 1A shows the expression of the differentiation marker keratin 10 (K10, red) in suprabasal cells, and Figure 1B shows the staining of basal keratinocytes and keratinocytes in the process of leaving the basal layer with an anti-keratin 15 (K15, red) antibody in human abdominal skin. In both figures, hemidesmosomes (which anchor the basal cells to the basement membrane) are visualized by means of the integrin β4 (iβ4) expression (green). Staining for the proliferation marker Ki67 revealed two groups of cycling keratinocytes that cluster (1) in the basal layer (white arrow heads in Figure 1C–E) and (2) in the first layers just above the *stratum basale* (arrows in Figure 1C–E).

For the exact quantification and localization of the two Ki67-positive cell populations, skin was digested and epidermal single cells were isolated, cytospinned on slides and immunofluorescence stained for K10, K15, iβ4 and Ki67 (Figure 1F). Proliferative suprabasal keratinocytes (Ki67^+^, K10^+^, iβ4^−^, arrow 1, Figure 1F) and proliferative basal keratinocytes (Ki67^+^, K15^+^, iβ4^+^, arrow 2) were identified among non-proliferative keratinocytes (arrows 3 and 4, compare also Figure S1). For keratinocytes freshly extracted from foreskins (Figure 1F’, left), we found that 83 ± 13% of all epidermal Ki67-positive cells resided in suprabasal layers, whereas only 17 ± 13% of the Ki67-positive cell pool were located in the basal layer during homeostasis. We obtained similar results with keratinocytes extracted from adult abdominal skin (Figure 1F’, right): 82 ± 14% of all epidermal Ki67-positive cells were found in suprabasal layers, whereas only 18 ± 14% of the Ki67-positive cell pool were located in the basal layer.

### 3.2. Sorting of Basal and Suprabasal Keratinocytes from Human Skin

Only a few extracellular surface markers allow for the discrimination of basal and suprabasal cells. We used the integrin α6β4 receptor (iα6β4) as the most popular for further investigations. We first confirmed the presence of integrin α6 high-expression (iα6^high^) and integrin α6 low-expression (iα6^low^) keratinocytes in the epidermis by FACS analyses (Figure 2A). In addition, we observed that the iα6^high^ population expressed iβ4, while the iα6^low^ population was negative for iβ4 (Figure 2A). The iα6^high^/iβ4^+^ cells represent the basal keratinocytes, whereas the iα6^low^/iβ4^−^ and the iα6^−^/iβ4^−^ cells represent the suprabasal keratinocyte population (Figure 2A).

Further, the FACS analysis confirmed the presence of two distinct, separable populations of interfollicular keratinocytes in the human epidermis: iβ4^+^/K10^−^ basal cells and iβ4^−^/K10^+^ suprabasal cells (Figure 2B). This finding correlates with the presence of iβ4^+^/K10^−^ basal cells and iβ4^−^/K10^+^ suprabasal cells shown in the histological analyses of human skin in Figure 1C–E.

We found the discrimination of basal and suprabasal keratinocytes to be more accurate using iβ4 than iα6 expression. Therefore, for sorting, we decided to use the iβ4 (Figure 2C). Figure 2D,E show the re-analysis of the samples after sorting which demonstrate the efficiency of the procedure. Sorted basal iβ4^+^ cells still contained approximately 1% of iβ4^−^ cells, whereas sorted suprabasal iβ4^−^ cells were pure.

We performed proteomics analysis to confirm the distinct protein expression in the sorted cell populations: expression of typical epidermal basal layer proteins such as K5 and K14, iα6, or collagen 17 were observed in basal iβ4^+^ cells, whereas suprabasal markers like K6b and Galectin-1 were found in the iβ4^−^ fraction (Figure S2). Melanocytes and Langerhans cells reside in the epidermis but are devoid of iβ4. They were sorted in the iβ4^−^ cell population. In fact, the iβ4^−^ cell population showed significant upregulation of the following proteins: CSC1-like protein 2 and Vimentin (both expressed in Langerhans cells) and melanoma antigen (expressed in melanocytes) (Figure S2).

### 3.3. Slow Adhering iβ4^−^ Cells Regain the Ability to Adhere and Proliferate in Culture

**Figure 3:**
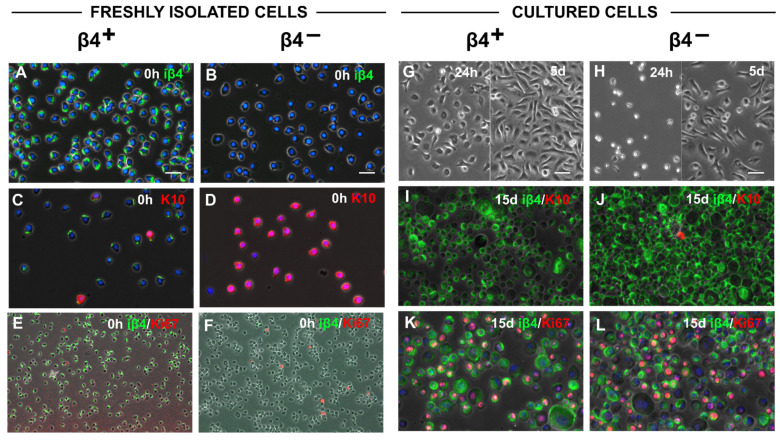
Both basal and suprabasal cells are activated during 2D culture in vitro. (**A**–**F**) Freshly isolated and iβ4-sorted keratinocytes were concentrated by cytospin and stained for different markers. (**A**,**B**) iβ4 staining (green) after sorting (0 h). iβ4^+^ cells were positive (**A**), iβ4^−^ were not (**B**). Nuclei were stained with Hoechst. (**C**,**D**) K10 staining (red) after sorting (0 h). iβ4^+^ cells were negative for K10 (**C**), with few exceptions (the residual green iβ4 signal is due the sorting procedure), iβ4^−^ were positive (**D**). Nuclei were stained with Hoechst. (**E**,**F**) Ki67 (red)/iβ4 (green) double-staining after sorting (0 h). More Ki67-positive cells were found in the iβ4^−^ fraction. (**G**–**L**) Freshly isolated and iβ4-sorted keratinocytes were cultured for two weeks before being removed from the culture plate, concentrated by cytospin and stained for different markers. (**G**,**H**) Phase-contrast pictures showing the morphology of cultured cells 24 h and 5 days after sorting. (**H**) iβ4^−^ keratinocytes went through an initial lag phase of proliferation (24 h) but recovered after a few days (5 d). (**I**,**J**) K10 (red)/iβ4 (green) staining after 15 days of culture: both iβ4+ (**I**) and originally iβ4^−^ cells (**J**) expressed now high levels of iβ4. (**K**,**L**) Ki67 (red)/iβ4 (green) double-staining after 15 days of culture: both iβ4^+^ (**K**) and originally iβ4^−^ cells (**L**) proliferated vigorously (Ki67, red) and were not distinguishable from each other any longer. Scale bars for all panels: 20 µm with exception of e and f (50 µm).

To elucidate the properties of sorted basal and suprabasal cells, we first compared adhesion and proliferation ability of iβ4^+^ and iβ4^−^ cells in vitro directly after isolation or after two weeks culture time. Figure 3A–F show the staining of keratinocytes, which were just isolated, sorted for iβ4^+^ and iβ4^−^ and then immediately centrifuged to a cytospin slide. As expected, at time point 0 h, exclusively basal (iβ4^+^) cells expressed iβ4 (green in Figure 3A,B) and only suprabasal (iβ4^−^) cells expressed K10 (red in Figure 3C,D), although we found a very low number of K10-positive basal cells (red cells in Figure 3C).

In line with the staining of natural (homeostatic) skin in Figure 1, at time point 0 h, the majority of the proliferative Ki67-expressing cells were found in the iβ4^−^ population (Figure 3E,F, red). However, during the first days in culture, iβ4^+^ keratinocytes displayed a higher adhesive and proliferative activity, whereas iβ4^−^ keratinocytes needed more time to adhere and start cell division (Figure 3G,H, 24 h). Only upon cultivation on dishes both iβ4^−^ and iβ4^+^ cells proliferated similarly well (Figure 3G,H, 5 d). After two weeks, K10 expression was lost and (originally) suprabasal iβ4^−^ cells started to express iβ4 (Figure 3I,J). The Ki67 expression showed that both iβ4^+^ and iβ4^−^ cell populations proliferated strongly at this time point in culture (Figure 3K,L, red).

### 3.4. Basal and Suprabasal Keratinocytes Lose Their Distinct Raman Spectra In Vitro

We analysed the biochemical–molecular properties of basal and suprabasal keratinocytes by Raman spectroscopy.

**Figure 4:**
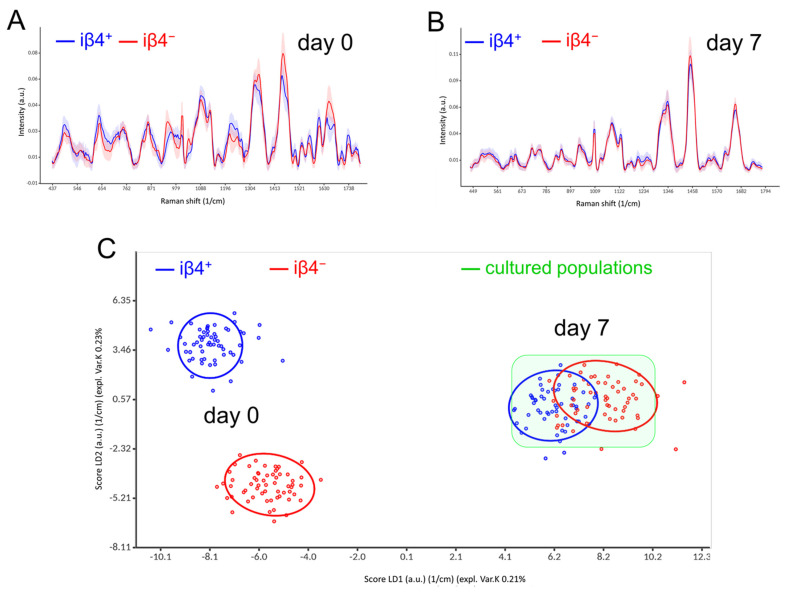
Raman spectroscopy suggests the relative phenotypical resemblance of basal and suprabasal cells after 2D culture. (**A**,**B**) Mean Raman spectra of iβ4+ (blue) and iβ4^−^ (red) cells just after sorting (**A**) or after 7 days of 2D culture (**B**). The shadings represent the standard deviation (*n* = 60). (**C**) Score plot of the Linear Discriminant Analysis (LDA) revealing two distinct populations at day 0 (left) and overall, two spectrally identical populations at day 7 (right), which both completely differ from the original populations. Each dot represents a Raman measurement, and the ellipses describe 90% confidence interval. The green region underlines the similarity of the cultured keratinocyte populations.

We collected Raman spectra from iβ4^+^ and iβ4^−^ keratinocytes, just after sorting (day 0) or after 7 days of culture (day 7). Raman mean spectra (*n* = 60) at day 0 showed distinctive variations in the biochemical composition between the two cell types (Figure 4A). Principal component analysis (PCA) was conducted on the Raman data. Since the first principal component (PC1) explains most of the variances in the data, the PC1-loadings plot (Supplementary Figure S3A) were used to display the spectral differences between the two cell types which are referred in part to the accumulation of lipids and fatty acids, like cholesterol, phosphatidylcholine, and ceramides in suprabasal cells (Supplemenatry Figure S3B) [40,41]. Examples are the increases of Raman bands at 1304, 1344, and 1445 cm^−1^ (C–H twisting and scissoring vibrations) in the case of suprabasal iβ4^−^ keratinocytes compared to iβ4^+^ basal keratinocytes (Supplementary Figure S3). The intensity of these bands increased by the higher numbers of CH groups consequently to the CH-chains of lipids and fatty acids [42].

Interestingly, no significant differences in the Raman bands between the two cell types were observed after 7 days of culture (Figure 4B). The Linear Discriminant Analysis (LDA) scores illuminate the clear differences in the Raman data of the 2 cell types at day 0 and the high similarity at day 7 (Figure 4C). To exclude the influence of the antibody staining needed for FACS, we applied hierarchical cluster analysis (HCA) on the Raman data of a fresh isolated unsorted sample. The spectra of the iβ4^+^ and iβ4^−^ keratinocyte sub-populations could be separated to two distinctive clusters similar to the Raman data acquired from the sorted keratinocytes (Supplementary Figure S4).

### 3.5. Slow Adhering iβ4^−^ Cells Regain the Ability to form Colonies in Culture

**Figure 5:**
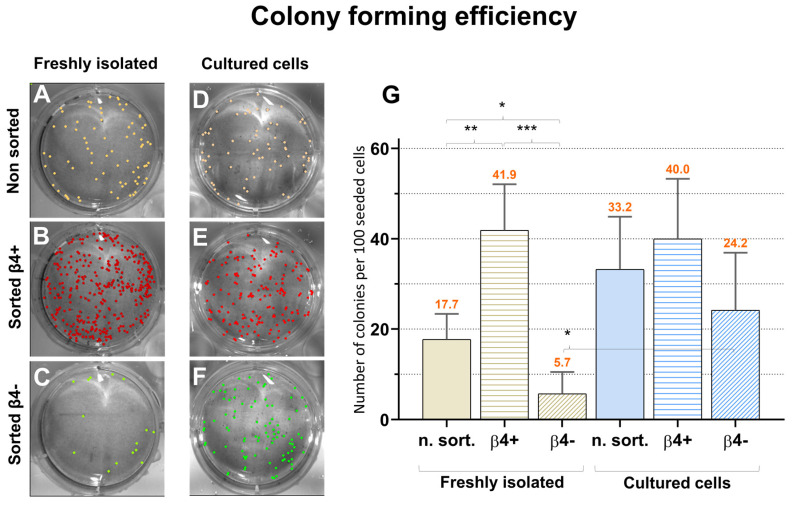
The difference in colony-forming efficiency between basal and suprabasal cells is reduced after 2D culture. (**A**–**C**) Freshly isolated keratinocytes were sorted and (**A**) 200 unsorted, (**B**) iβ4^+^, or (**C**) iβ4^−^ cells were plated separately on a feeder layer of 3T3-Swiss albino mouse fibroblasts. After 10 days, the formed colonies were visualized by trypan blue staining and counted. (**D**–**F**) The same was performed with (**D**) unsorted, (**E**) iβ4^+^ or (**F**) iβ4^−^ cells which were previously cultured for 15 days on collagen type I. The statistical analysis (**G**) shows the number of colonies formed per 100 seeded cells (means of 8 experiments, standard deviations, and significances from the unpaired *t* test: * *p* < = 0.0037, ** *p*= 0.000547, *** *p*= 0.000001, n.S = *p* > 0.05), and indicates that after 2D culturing basal and suprabasal cells own similar colony-forming efficiencies.

We also compared the colony-forming efficiency of unsorted keratinocytes and sorted iβ4^+^ and iβ4^−^ keratinocytes. The keratinocytes of eight different donors were sorted and analysed as follow: Equal number of keratinocytes were plated (unsorted, iβ4^+^ or iβ4^−^) then 10 days after seeding, the number of formed colonies was counted (Figure 5A–C). Alternatively, the cells were first cultured for 15 days before the colony-forming assay was performed (Figure 5D,F). Figure 5G summarizes the results: Freshly isolated basal iβ4^+^ cells formed significantly more colonies (41.9% ± 10.2) than suprabasal iβ4^−^ cells (5.7% ± 4.8). Yet, after pre-culturing for 15 days, significantly more iβ4^−^ cells were able to produce a colony (24.2% ± 12.7), so that the difference to iβ4^+^ cells was not significant anymore (*p* > 0.05). Unsorted cells, as a mixture of both cell types, produced always intermediate results (17.7% ± 5.7 and 33.2% ± 11.7).

### 3.6. Basal and Suprabasal Keratinocytes Are Competent for Ensuring the Long-Term Survival of Human Dermo–Epidermal Skin Substitutes In Vivo

**Figure 6:**
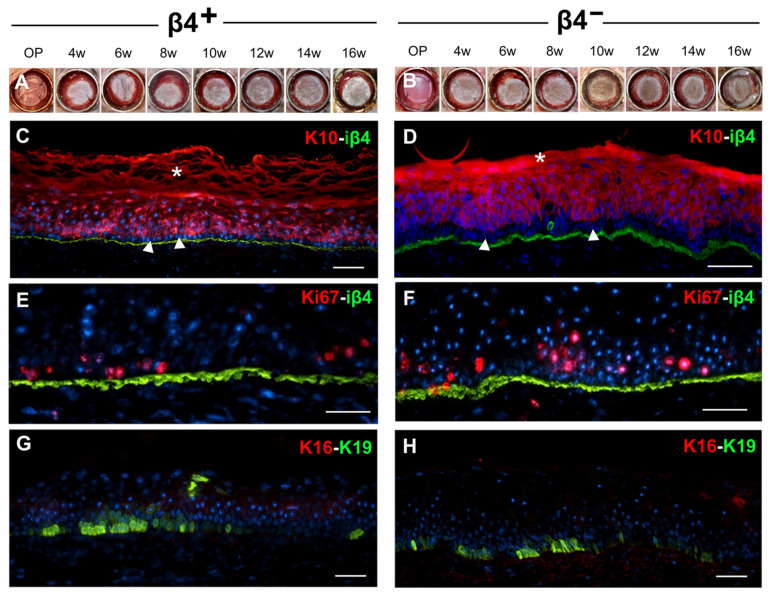
Basal and suprabasal keratinocytes show equal regeneration potential in vivo. Freshly isolated and sorted iβ4^+^ and iβ4^−^ keratinocytes were separately included in dermo–epidermal skin substitutes and transplanted on nude immuno-incompetent rats. (**A**,**B**) Macroscopic view of the iβ4^+^ (**A**) and iβ4^−^ (**B**) transplants, respectively, during 16 weeks in vivo. Thereafter the grafts were sectioned and analysed by immunofluorescence staining for (**C**,**D**) human basement membrane iβ4 (green) and suprabasal differentiation marker human K10 (red, * denotes unspecific staining in the cornified layer), (**E**,**F**) iβ4 (green) and proliferation marker Ki67 (red), (**G**,**H**) human basal K19 and would healing marker K16 (red). White stars: unspecific staining in the stratum corneum. Scale bar: 50 µm.

Since both iβ4^+^ and iβ4^−^ keratinocytes displayed high proliferative and colony-forming properties, we investigated whether they would develop into a multi-layered stratified epidermis in vivo. Hence, we used freshly isolated and sorted iβ4^+^ or iβ4^−^ cells (which were never in contact with culture plastic) to prepare dermo–epidermal skin equivalents which were transplanted and maintained on the backs of immune-incompetent rats for up to 16 weeks. We observed that both iβ4^+^ and iβ4^−^ keratinocytes had equal capacity to develop into a stratified epidermis. Figure 6A,B show a representative experiment of a total of three transplantations per cell type.

Immunofluorescence staining for keratin 10 (K10) revealed the presence of a K10-negative basal layer in both iβ4+ and iβ4^−^ constructs (Figure 6C,D, arrowheads). Furthermore, the expression of the hemidesmosomal iβ4 (green, Figure 6C–F) and its ligand in the basement membrane Laminin 332 (Lam332, red, Figure S5C,D), as well as the basal markers K19 (green, Figure 6G,H) and K15 (green, Figure S5A,B), revealed the presence of a differentiated basal layer and a dermo–epidermal junction. Moreover, ordered stratification was visible using immunofluorescence staining against cytokeratin 2e (K2e, green) in a spinous and granular layer (Figure S5C,D), desmoglein 3 (Dsg3, red) in the spinous layer and desmoglein 1 (Dsg1, green) in the granular layer (Figure S5E,F). Ki67 staining (red) revealed the typical distribution of dividing cells mainly in the first suprabasal layers in the transplanted skin substitutes (Figure 6E,F).

To investigate whether there were signs of wound healing, we stained the samples with an antibody against K16. All epidermal substitutes constituted by cultured iβ4^+^ or iβ4^−^ keratinocytes did not express K16 anymore after 16 weeks of transplantation (Figure 6G,H), indicating an advanced homeostasis.

## 4. Discussion

Histological staining of human skin which showed suprabasal keratinocytes as the main proliferative keratinocyte population was the starting point of this study. This observation conforms with earlier published data [9,10,11], as well with a few recent works [43,44]. However, it is in contrast to many other studies, which proposed that proliferating transit amplifying cells and quiescent keratinocyte stem cells (SCs) co-exist only in the basal cell layer [2,12,45]. In fact, this contradiction is caused from data acquired in mice, and uses the murine system as the representative mammalian system, thus, extrapolating to humans. In mice, suprabasal cycling keratinocytes are very rare in the epidermis [12] and a relevant pool of proliferating keratinocyte (stem cells) may be located in the numerous hair follicles that constitute the fur [13,46].

Acknowledging the discrepancies between mice and human observations, we focused our investigation on human skin (young foreskin and adult abdominal interfollicular skin). We sorted two keratinocyte populations according to the expression of the iβ4 chain. iβ4 forms heterodimers with iα6 (iα6, CD49f) which in turn is a biomarker commonly found in more than 30 different populations of SCs [47]. The iα6β4 receptor has been shown to be an important marker of epithelial, myogenic stem, and progenitor cells [12,45,48,49,50,51], and even to be upregulated in keratinocyte SCs [23]. We show in our study that basal iα6^high^ keratinocytes are exclusively positive for iβ4, whereas the suprabasal iα6^low^ keratinocyte population express the differentiation markers K1 and K10.

Some previous in vitro studies on the human epidermis showed that iβ4^+^ keratinocytes are fast cycling, and iβ4^−^ cells are post-mitotic differentiating keratinocytes with limited proliferative ability [52,53]. Our experiments confirmed this observation: although largely quiescent in their physiological situation, basal iβ4^+^ keratinocytes are induced to vigorously proliferate after their dissociation into single cells and culture in vitro. In contrast, we show here that suprabasal iβ4^−^ keratinocytes (manifestly more proliferative in homeostatic human epidermis but devoid of basement membrane adhesion molecules) needed two weeks to acquire a similar proliferative capacity as basal iβ4^+^ keratinocytes in vitro. In this period of time, terminally differentiated cells (possibly granular and upper spinous cells) may not adhere to the cell culture dish, introducing a selective enrichment of those early differentiated suprabasal keratinocytes which maintain a potential to de-differentiate back to basal stem cells. This may happen both during the 2D-culture on plastic dishes before performing proliferation, colony-forming and Raman spectral analyses and during the 3D-organotypic culture in vitro before transplantation. Due to the high purity of the sorting procedure shown in Figure 2 we consider it unlikely that contaminating iβ4^+^ keratinocytes overgrew the culture of sorted iβ4^−^ keratinocytes in the short time before in vitro analyses or before transplantation. Also, we did not observe any particular cell dying in the sorted, plated, and adherent iβ4^−^ keratinocytes in the 2D-cultures. We assume instead that iβ4^−^ keratinocytes pass through a re-programming phase in the culture (both in 3D-skin substitutes and 2D-culture on plastic), which includes the activation of the genes for integrin receptors and, conceivably, other (adhesion) molecules and the deactivation of genes for differentiation proteins. Yet, once the proliferation is triggered, both isolated and cultured basal and suprabasal keratinocytes (1) express high levels of iβ4 and downregulate K10 expression, (2) demonstrate similar colony-forming efficiencies, (3) express analogous phenotype by displaying similar Raman spectral fingerprints, (4) establish a basement membrane and a functional niche, and, finally and most important, (5) both iβ4^+^ and iβ4^−^ keratinocytes develop into a epidermis in skin substitutes, which is maintained for at least 16 weeks in a pre-clinical animal model. Therefore, based on these results, we believe to observe that in normal interfollicular skin, the biological (marker expression, colony-forming efficiency, and transplantation) and biochemical (Raman fingerprint) properties of basal and suprabasal cells after culture in vitro become similar, which may reflect a similar regenerative potential.

**Figure 7:**
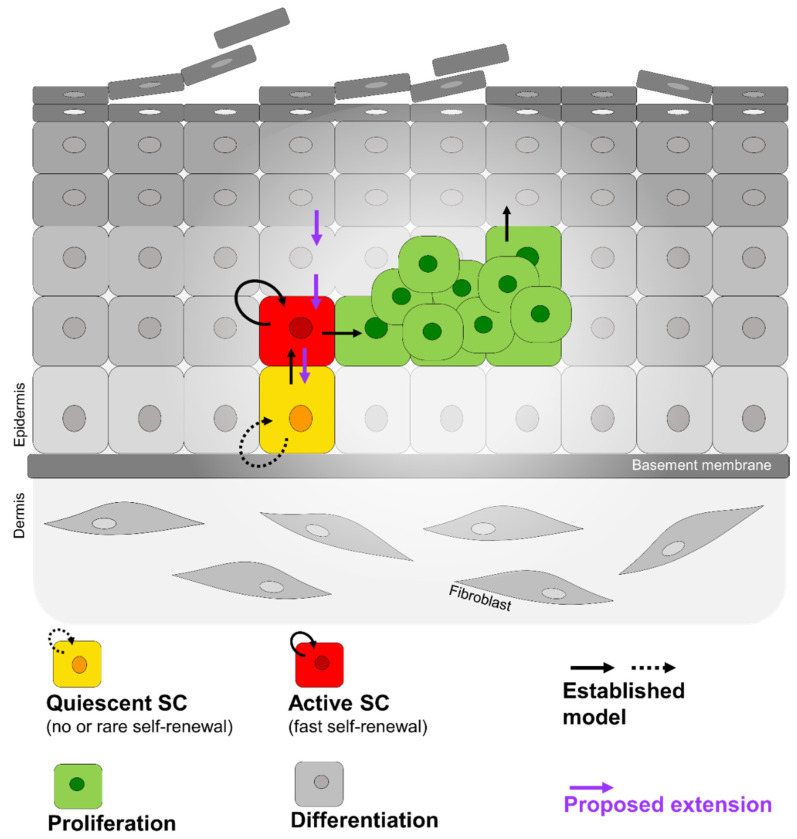
Quiescent and active stem cells assure epidermal self-renewal in human skin. Quiescent stem cells (yellow) residing in the basal layer of human interfollicular epidermis replace lost active self-renewing stem cells (red) present in the suprabasal layers. The active stem cell progeny (green) proliferate and differentiate to replenish the suprabasal keratinocyte layers. In addition, we propose that in a wound situation or triggered by the contact with the basement membrane, suprabasal not yet irreversibly differentiated keratinocytes (grey) can retro-differentiate into active or even quiescent SC (violet arrows).

Thus, we speculate that two distinct keratinocyte SC populations may exist in the human interfollicular epidermis, a quiescent (rarely cycling) one in the basal layer and an active (fast cycling) one in the suprabasal compartment. Consequently, we postulate that K10^+^ iβ4^−^ suprabasal keratinocytes can retro-differentiate into active or even quiescent SC when they face a wound situation and/or when they get into contact with the basement membrane (Figure 7).

Interestingly, Schlüter et al. [54] analysed in humans the molecular profile of quiescent keratinocyte SC (iα6^bri^ CD71^dim^, in our work iβ4^+^), transit amplifying (iα6^bri^ CD71^bri^, in our work also iβ4^+^) and early differentiating (iα6^dim^, in our work iβ4^−^) keratinocyte populations and evaluated them in transplantation experiments. They observed a gradually dropping regenerative capacity and conceived of a continuum rather than a black and white transition between the keratinocyte stem and differentiating cells. The authors therefore speculate on the existence of cells with varying degrees of self-renewing capacities [54] which corresponds with our observations.

Similarly, after single cell-RNA sequencing, Wang et al. described four spatially different SC populations located at the top or in the tip of rete ridges in the basal epidermal layer [55]. They suggest these SC populations to represent basal SC with an “unstable” (i.e., still undefined or fluid) cell fate in the course of leaving the basal layer. The authors oppose the theory of a single population of SCs and suggest the existence of multiple epidermal SC levels in the basal layer, which contribute to the maintenance of epidermal homeostasis. Our data expand this concept to the suprabasal layers of the human epidermis.

In fact, both studies [54,55] do not deviate from the hypothesis of the absolute irreversibility of the differentiation process, and consider suprabasal keratinocytes as definitively committed cells. In opposition to that, our data suggest that “stemness” in suprabasal cells may be continuously fading towards the stratum granulosum but not in an absolute irreversible way.

In support of this hypothesis, some other authors found indications of a possible de-differentiation capacity of committed suprabasal keratinocytes which would allow them to re-acquire “stemnes” properties: Fu et al. treated patients that had leg ulcers with topical recombinant human epidermal growth factor and observed the induction of expression of the stem cell marker K19 in the spinous and granular layer of the regenerated epidermis [56]. Similarly to us, Mannik et al. showed in mice that post-mitotic differentiated keratinocytes were able, after a culturing period, to reform a self-renewing, hair-bearing skin in vivo, demonstrating that commitment to differentiation does not exclude the possibility of “re-entering the cell cycle, de-differentiating, and acquiring stemness’’ [57]. Further, Rampolas et al. showed that a stem cell niche (in this case the laser-ablated bulge region of hair follicles) can induce the reprogramming of committed cells for replacing the depleted stem cell pool [58]. Based on these findings, Chacón-Martínez et al. defined a set of factors that can drive this reprogramming [59]. More recently, single-cell RNA sequencing profile analysis of mouse cells from different epidermal layers during wound regeneration revealed indications for a bidirectional cell fate fluidity between basal and spinous cells [60].

Trying to find a synthesis of many observations made in different tissues, such as the gut, hair follicle, bone marrow, and brain, Li et al. proposed a general model which implies the existence of two stem cell populations, referred to as quiescent and active stem cells, being located in separate yet adjoining locations, the basal and the suprabasal compartment, respectively [22,61]. They suggested that a certain number of damaged, active stem cells may constantly be replaced by quiescent stem cells. The authors state “This would prevent the active stem cell pool from becoming exhausted and protect against potentially tumorigenic mutations. Conversely, active stem cells may have the capacity to replace lost or damaged quiescent stem cells” under certain specific circumstances [22].

We propose that this could also be true for the human epidermis (Figure 7), even though in our assay the grafted cells may have undergone at first dedifferentiation in the organotypic culture before transplantation (similar to the cells in 2D-culture). Further, we did not find indications that each of the two self-renewing keratinocyte types (quiescent and active SCs) can transform into its counterpart under the described experimental circumstances. Other important questions which cannot be inferred from our data are, where to set the point of no return (if there is one) in the epidermal differentiation process beyond which the self-renewal ability is not guaranteed anymore, and which mechanisms may be involved.

## 5. Conclusions

In conclusion, our data show that basal and suprabasal keratinocytes own self-renewing potential and, after 3D-organotypic or 2D cultivation in vitro, can generate and maintain a stratified epidermis for many weeks in vivo. We suggest that the interplay between basal quiescent and suprabasal active keratinocytes stem cells is part of a self-renewal system for the maintenance and damage repair of the adult human interfollicular epidermis during homeostasis.

## Data Availability

The data underlying this article will be shared on reasonable request to the corresponding author. Raw mass spectrometry proteome data and search results have been deposited to the ProteomeXchange Consortium (http://proteomecentral.proteomexchange.org, accessed on 13 June 2022) via the Proteomics Identifications Database (PRIDE) partner repository with the data set identifier PXD034543.

## Supplementary Materials

The following supporting information can be downloaded at: https://www.mdpi.com/article/10.3390/cells11142156/s1, Figure S1: Staining of freshly isolated keratinocytes; Figure S2: Proteomics analysis of sorted basal and suprabasal keratinocytes.; Figure S3: Spectral differences between freshly isolated and sorted iβ4^+^ and iβ4^−^ keratinocytes.; Figure S4: Hierarchical cluster analysis on the Raman data of freshly isolated unsorted keratinocytes shows two distinctive clusters similar to data acquired from the sorted cells.; Figure S5: Quality of substitutes produced with iβ4^+^ and iβ4^−^ keratinocytes.
