# Supplementary material for: Human Basal and Suprabasal Keratinocytes Are Both Able to Generate and Maintain Dermo–Epidermal Skin Substitutes in Long-Term In Vivo Experiments
Source: Cells. 2022 Jul 9;11(14):2156. doi: 10.3390/cells11142156 (PMC9319791; doi:10.3390/cells11142156)
Supplement: Supplementary file 1 [file cells-11-02156-s001.zip › cells-1794375-supplementary.pdf]

Supplementary Figures

Back Matter

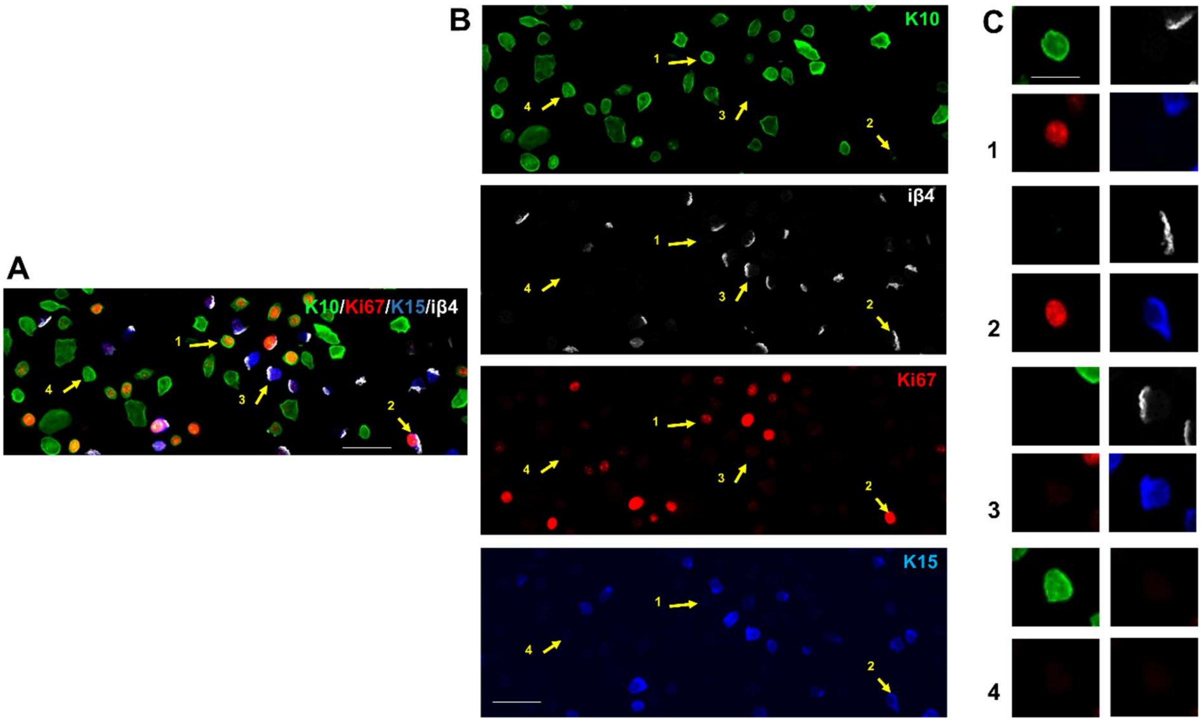

Figure S1. Staining of freshly isolated keratinocytes. **(A)** Copy of Fig. 1F: Cytospin centrifugation and staining of cells for Ki67 (red) and K10 (green), K15 (blue) and iβ4 (white). Yellow arrows point on four different cell types: proliferating suprabasal cells (1), proliferating basal cell (2), quiescent basal cell (3) and quiescent suprabasal cell (4). **(B)** Splitting of the color channels. **(C)** Magnification of the pointed cells. Scale bar in A,B 50 μm, in C 25 μm.

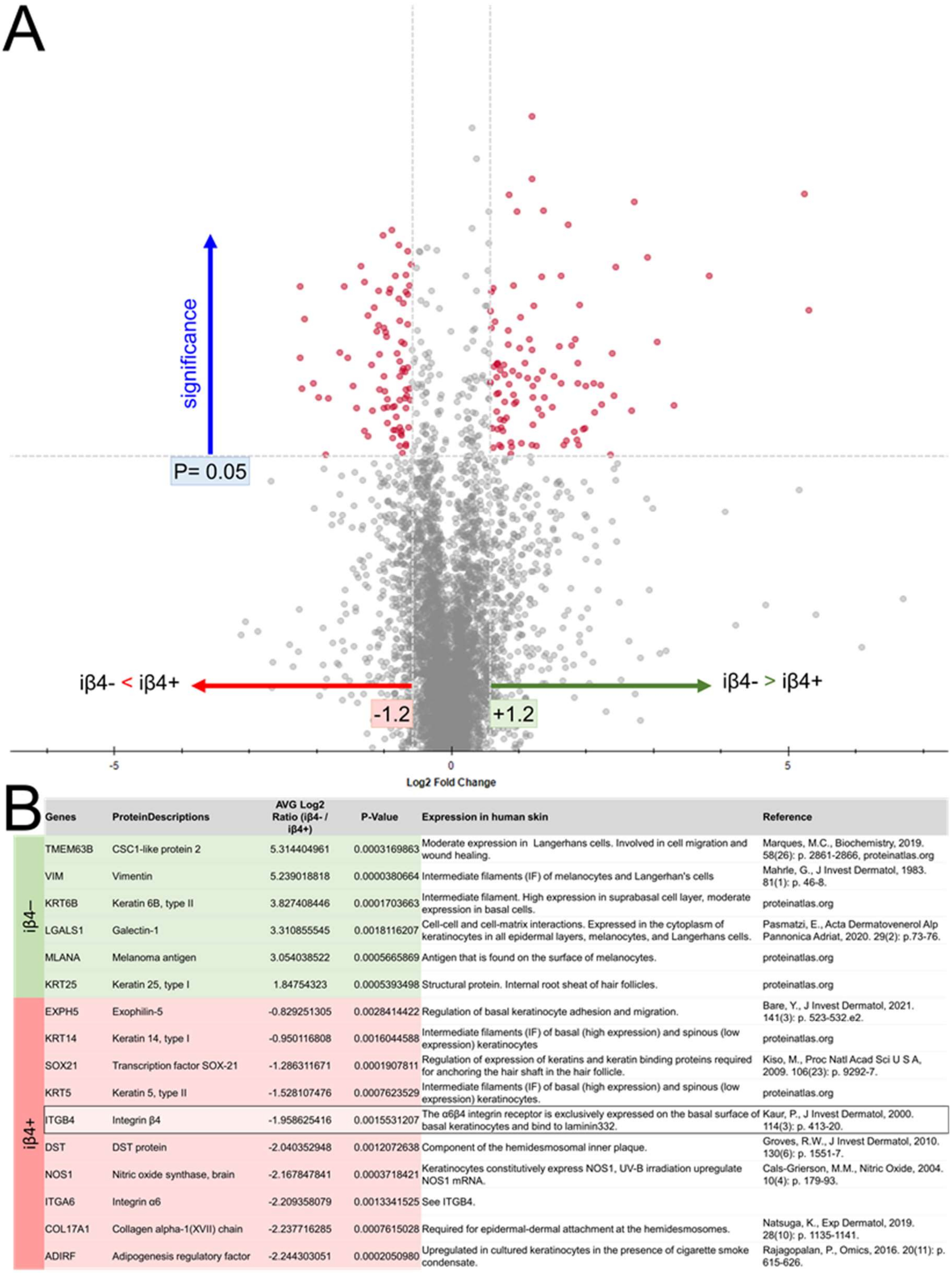

B

Group	Genes	ProteinDescriptions	AVG Log2 Ratio (iβ4- / iβ4+)	P-Value	Expression in human skin	Reference
iβ4−	TMEM63B	CSC1-like protein 2	5.314404961	0.0003169863	Moderate expression in Langerhans cells. Involved in cell migration and wound healing.	Marques, M.C., Biochemistry, 2019. 58(26): p. 2861-2866, proteinatlas.org
iβ4−	VIM	Vimentin	5.239018818	0.0000380664	Intermediate filaments (IF) of melanocytes and Langerhan's cells	Mahrle, G., J Invest Dermatol, 1983. 81(1): p. 46-8.
iβ4−	KRT6B	Keratin 6B, type II	3.827408446	0.0001703663	Intermediate filament. High expression in suprabasal cell layer, moderate expression in basal cells.	proteinatlas.org
iβ4−	LGALS1	Galectin-1	3.310855545	0.0018116207	Cell-cell and cell-matrix interactions. Expressed in the cytoplasm of keratinocytes in all epidermal layers, melanocytes, and Langerhans cells.	Pasmatzi, E., Acta Dermatovenerol Alp Pannonica Adriat, 2020. 29(2): p.73-76.
iβ4−	MLANA	Melanoma antigen	3.054038522	0.0005665869	Antigen that is found on the surface of melanocytes.	proteinatlas.org
iβ4−	KRT25	Keratin 25, type I	1.84754323	0.0005393498	Structural protein. Internal root sheat of hair follicles.	proteinatlas.org
iβ4+	EXPH5	Exophilin-5	-0.829251305	0.0028414422	Regulation of basal keratinocyte adhesion and migration.	Bare, Y., J Invest Dermatol, 2021. 141(3): p. 523-532.e2.
iβ4+	KRT14	Keratin 14, type I	-0.950116808	0.0016044588	Intermediate filaments (IF) of basal (high expression) and spinous (low expression) keratinocytes	proteinatlas.org
iβ4+	SOX21	Transcription factor SOX-21	-1.286311671	0.0001907811	Regulation of expression of keratins and keratin binding proteins required for anchoring the hair shaft in the hair follicle.	Kiso, M., Proc Natl Acad Sci U S A, 2009. 106(23): p. 9292-7.
iβ4+	KRT5	Keratin 5, type II	-1.528107476	0.0007623529	Intermediate filaments (IF) of basal (high expression) and spinous (low expression) keratinocytes.	proteinatlas.org
iβ4+	ITGB4	Integrin β4	-1.958625416	0.0015531207	The α6β4 integrin receptor is exclusively expressed on the basal surface of basal keratinocytes and bind to laminin332.	Kaur, P., J Invest Dermatol, 2000. 114(3): p. 413-20.
iβ4+	DST	DST protein	-2.040352948	0.0012072638	Component of the hemidesmosomal inner plaque.	Groves, R.W., J Invest Dermatol, 2010. 130(6): p. 1551-7.
iβ4+	NOS1	Nitric oxide synthase, brain	-2.167847841	0.0003718421	Keratinocytes constitutively express NOS1, UV-B irradiation upregulate NOS1 mRNA.	Cals-Grierson, M.M., Nitric Oxide, 2004. 10(4): p. 179-93.
iβ4+	ITGA6	Integrin α6	-2.209358079	0.0013341525	See ITGB4.	
iβ4+	COL17A1	Collagen alpha-1(XVII) chain	-2.237716285	0.0007615028	Required for epidermal-dermal attachment at the hemidesmosomes.	Natsuga, K., Exp Dermatol, 2019. 28(10): p. 1135-1141.
iβ4+	ADIRF	Adipogenesis regulatory factor	-2.244303051	0.0002050980	Upregulated in cultured keratinocytes in the presence of cigarette smoke condensate.	Rajagopalan, P., Omics, 2016. 20(11): p. 615-626.

Figure S2. Proteomics analysis of sorted basal and suprabasal keratinocytes. Mass spectrometric analysis (DIA) of protein extracts from freshly isolated and sorted keratinocytes ($i\beta4^{+}$ or $i\beta4^{-}$) **(A)** Volcano plot showing proteomics data. These points indicate different proteins that display both large magnitude fold-changes (x-axis) and high statistical significance (-log10 of p values, y-axis). Dashed horizontal line shows the p values cut-off (p=0.05) and the two vertical dashed lines indicate down/up regulated proteins. **(B)** A partial list of overexpressed proteins in $i\beta4^{+}$ and $i\beta4^{-}$ keratinocytes.

A

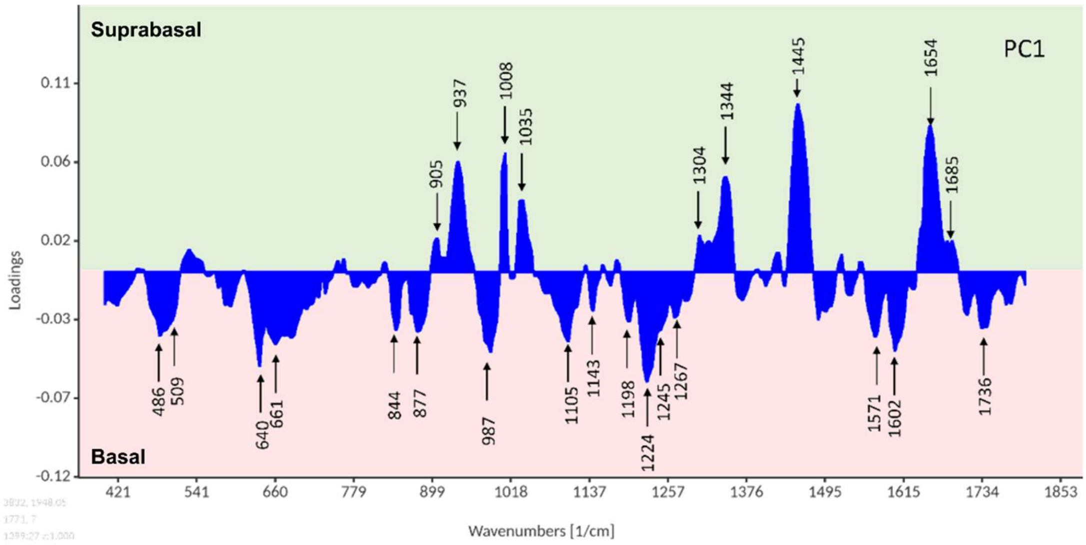

B

Peak	Assignment	iβ4	Epidermal compartment
**905**	Mono and di-saccharide	-	**Suprabasal**
**937**	Proline	-	
**1008**	Phenylalanine	-	
**1035**	Collagen	-	
**1304**	Lipids	-	
**1344**	Lipids, Proteins	-	
**1445**	Cholesterol, fatty acids, proteins	-	
**1643**	Protein	-	
**1654-1685**	Cholesterol, ceramides, proteins	-	
**486**	Glycogen	+	**Basal**
**509**	Disulfide-protein	+	
**640**	Tyrosine, protein	+	
**661**	Cystein, Collagen	+	
**844-877**	Collagen, Proteins	+	
**987**	Proteins	+	
**1105**	Fatty acids, Proteins	+	
**1143**	Fatty acids	+	
**1198**	Tyrosine, protein	+	
**1224-1267**	Collagen, protein	+	
**1602**	Phenylalanine, proteins	+	
**1736**	Lipid	+	

Figure S3. Spectral differences between freshly isolated and sorted iβ4+ and iβ4− keratinocytes. **(A)** PCA loading plot of PC1 indicating major spectral variation between the data sets of freshly isolated and sorted iβ4+ and iβ4− keratinocytes, in which positive bands describe intensity-increased Raman features of suprabasal and negative bands intensity-increased Raman features of basal cells. **(B)** Table of assignments of Raman spectral variations indicated in the PC1 loadings (A).

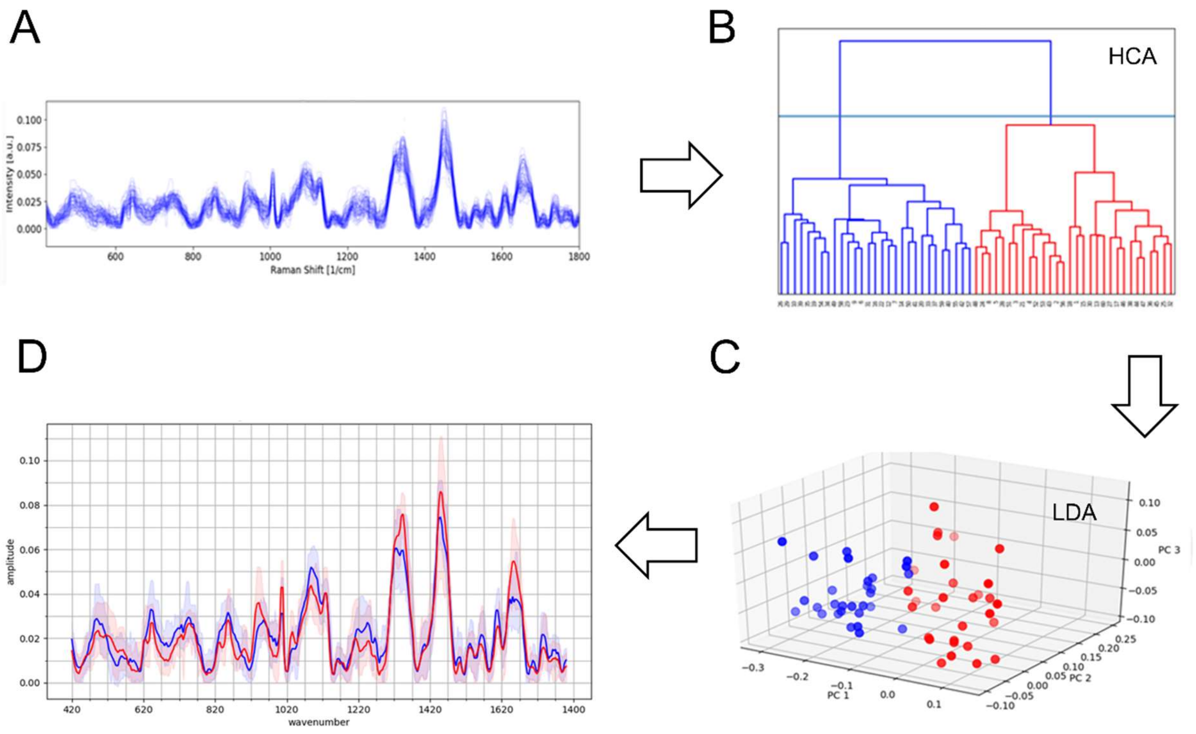

Figure S4. Hierarchical cluster analysis on the Raman data of freshly isolated unsorted keratinocytes shows two distinctive clusters similar to data acquired from the sorted cells. To exclude the influence of the antibody staining needed for FACS on the Raman fingerprint, the mean spectrum (n=60) of the **(A)** untreated, non-sorted population (containing basal and suprabasal cells) was subjected to **(B)** Hierarchical Cluster analysis (HCA) and **(C)** Linear Discriminant Analysis (LDA). **(D)** We obtained two distinguishable cell populations (blue and red), whose mean spectra were almost identical to the mean spectra of the stained and sorted populations (see in Fig. 4A).

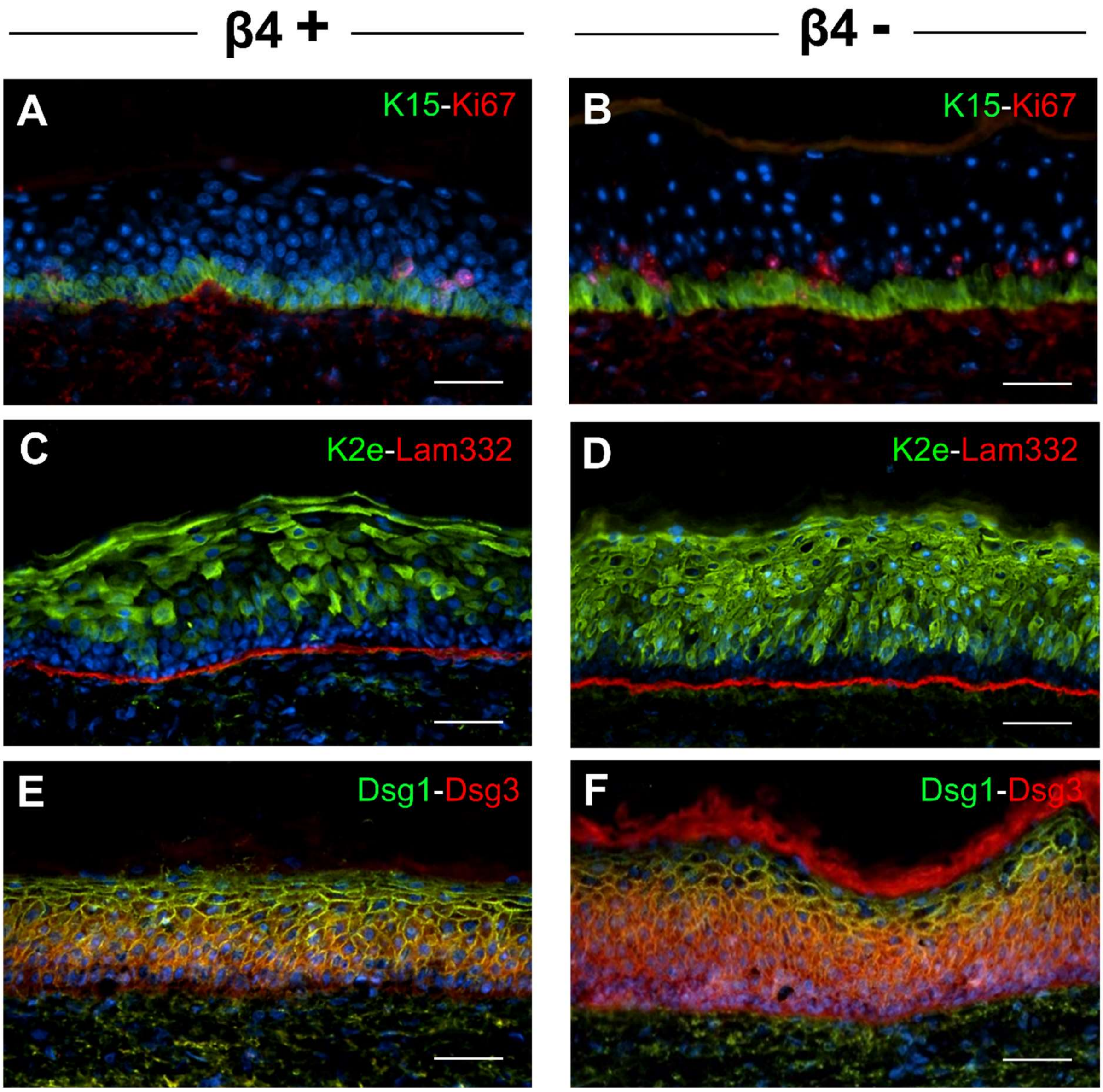

Figure S5. Quality of substitutes produced with iβ4+ and iβ4− keratinocytes.Sorted and cultured iβ4+ and iβ4− keratinocytes were separately included in dermo-epidermal skin substitutes and transplanted on nude rats for 16 weeks. Thereafter the grafts were sectioned and analysed by immunofluorescence staining for **(A-B)** basal K15 (green) and proliferation marker Ki-67 (red), **(C-D)** suprabasal differentiation marker K2e (green) and basal lamina component Laminin 332 (red), **(E-F)** suprabasal differentiation markers Desmoglein 1 (green) and Desmoglein 3 (red). No difference is visible between iβ4+ and iβ4− substitutes. White stars: stratum corneum. Scale bar: 50 μm.
